# DNA damage in blood leucocytes of prostate cancer patients during therapy with ^177^Lu-PSMA

**DOI:** 10.1007/s00259-019-04317-4

**Published:** 2019-04-27

**Authors:** Sarah Schumann, Harry Scherthan, Constantin Lapa, Sebastian Serfling, Razan Muhtadi, Michael Lassmann, Uta Eberlein

**Affiliations:** 1 Department of Nuclear Medicine, University of Würzburg, Oberdürrbacher Str. 6, 97080 Würzburg, Germany; 2 Bundeswehr Institute of Radiobiology affiliated to the University of Ulm, Neuherbergstr. 11, 80937 Munich, Germany

**Keywords:** DNA double-strand breaks, γ-H2AX, 53BP1, Biodosimetry, Absorbed dose to the blood, Prostate cancer, ^177^Lu-PSMA

## Abstract

**Purpose:**

The aim of this study was to investigate the time- and dose-dependency of DNA double-strand break (DSB) induction and repair in peripheral blood leucocytes of prostate cancer patients during therapy with ^177^Lu-PSMA.

**Methods:**

Blood samples from 16 prostate cancer patients receiving their first ^177^Lu-PSMA therapy were taken before and at seven time-points (between 1 h and 96 h) after radionuclide administration. Absorbed doses to the blood were calculated using integrated time–activity curves of the blood and the whole-body. For DSB quantification, leucocytes were isolated, fixed in ethanol and immunostained with γ-H2AX and 53BP1 antibodies. Colocalizing foci of both DSB markers were manually counted in a fluorescence microscope.

**Results:**

The average number of radiation-induced foci (RIF) per cell increased within the first 4 h after administration, followed by a decrease indicating DNA repair. The number of RIF during the first 2.6 h correlated linearly with the absorbed dose to the blood (*R*^2^ = 0.58), in good agreement with previously published in-vitro data. At late time-points (48 h and 96 h after administration), the number of RIF correlated linearly with the absorbed dose rate (*R*^2^ = 0.56). In most patients, DNA DSBs were repaired effectively. However, in some patients RIF did not disappear completely even 96 h after administration.

**Conclusion:**

The general pattern of the time- and dose-dependent induction and disappearance of RIF during ^177^Lu-PSMA therapy is similar to that of other radionuclide therapies.

## Introduction

Membrane glycoprotein prostate-specific membrane antigen (PSMA) is highly overexpressed in prostate cancer and metastases, making it a particularly suitable target for imaging and therapeutic agents [1, 2]. For the last 5 years, PSMA-binding ligands labelled with the β-particle-emitter ^177^Lu have been used in an increasing number of medical centres worldwide for the treatment of metastatic castration-resistant prostate cancer. Various studies have shown promising results including high response rates, a favourable safety profile and reduction in pain [3–8]. However, the ionising radiation from ^177^Lu not only destroys malignant tissue, but also leads to DNA damage in healthy cells. In particular, the haematopoietic system is an organ-at-risk for targeted radionuclide therapies. As the absorbed dose to the blood can be used as a surrogate marker for the absorbed dose to the bone marrow, the quantification of radiation-induced DNA damage in blood and its correlation with the absorbed dose to the blood is of great interest [9].

Among the different types of DNA damage, double-strand breaks (DSBs) are the most critical lesions, since they are difficult to repair, and misrepair can lead to mutations or cell death. DNA DSBs can be visualized by immunofluorescence staining with antibodies for γ-H2AX and 53BP1 [10–12]. The microscopically visible colocalizing γ-H2AX + 53BP1 foci in the cell nuclei can then be quantified to assess DNA DSB damage. Since these foci appear with the induction of DNA DSBs and disappear with the progression of DSB repair, they can be used to describe the time-dependency of DNA damage induction and repair during irradiation [13–16].

So far, only a few studies have investigated radiation-induced DNA damage during radionuclide therapy by quantifying γ-H2AX foci or colocalizing γ-H2AX + 53BP1 foci. Three studies have addressed focus formation during radioiodine therapy [9, 15, 17]. Two studies analysed the kinetics of focus formation during peptide receptor radionuclide therapy with ^177^Lu-labelled DOTA-d-Phe-Tyr3-octreotate (^177^Lu-DOTATATE) and ^177^Lu-labelled DOTA-d-Phe-Tyr3-octreotide (^177^Lu-DOTATOC) [14, 18]. Despite the increasing number of radionuclide therapies, DNA damage in blood leucocytes of patients during therapy with PSMA-binding ligands has not been investigated until now.

Therefore, the aim of the present study was to investigate the time- and dose-dependency of DSB induction and repair in blood leucocytes of prostate cancer patients during their first therapy cycle with ^177^Lu-PSMA.

## Materials and methods

### Patients

The study included 16 prostate cancer patients (P1–P16) receiving their first treatment with ^177^Lu-PSMA. A standard activity of 6 GBq of a PSMA ligand (PSMA I&T; SCINTOMICS GmbH, Munich, Germany) labelled with ^177^Lu (EndolucinBeta®; ITM Isotopen Technologien München AG, Munich, Germany) was intravenously administered over 20 min. The end of the administration process was chosen as the starting and reference point for this study. The patients were hospitalized for 2 days after the start of the treatment. All patients underwent a diagnostic PET/CT scan with a ^68^Ga-labelled PSMA ligand for uptake detection and staging/restaging of the disease and a MAG3 scan with ^99m^Tc before and after therapy to assess kidney function. Serum PSA levels were also determined before and after therapy. Therapy response was evaluated based on the imaging results, according to the modified PET Response Criteria in Solid Tumors (mPERCIST) and RECIST1.1 [19, 20], and changes in serum PSA levels, in analogy to the PCWG3 criteria [21].

### Blood sampling

Blood samples were taken prior to administration (*t*_0_; for determination of the individual number of baseline foci) and nominally at 1 h (*t*_1_), 2 h (*t*_2_), 3 h (*t*_3_), 4 h (*t*_4_), 24 h (*t*_5_), 48 h (*t*_6_) and 96 h (*t*_7_) after administration. Blood was drawn using lithium-heparin blood-collecting tubes (S-Monovette®; Sarstedt, Nümbrecht, Germany). Due to variations in the management of the individual patients, there were occasional deviations from the nominal sampling time-points. The last blood sample (96 h after administration) was missing in patients P5, P8 and P12. In patient P11, the *t*_3_ sample could not be evaluated.

### Activity quantification

An aliquot of each blood sample was measured in a calibrated high-purity germanium detector (Canberra, Rüsselsheim, Germany). The counting efficiency of the detector was ascertained by measuring several NIST- and NPL-traceable standards. For activity quantification, the γ-emission line of ^177^Lu at 208.4 keV (emission probability of 10.4%) was evaluated. The measured number of counts was decay-corrected to the start time of the measurement, and the corresponding activity value was then decayed to the time-point of the blood sampling.

### Evaluation of DNA damage: γ-H2AX + 53BP1 assay

The blood samples were processed and the γ-H2AX + 53BP1 foci evaluated following the protocol described by Eberlein et al. [22]. Briefly, leucocytes were separated by density centrifugation in BD Vacutainer CPT tubes (BD, Heidelberg, Germany), washed, fixed in 70% ethanol and stored at −20 °C. For immunofluorescence staining and focus analysis [23], the samples were shipped to the Bundeswehr Institute of Radiobiology in Munich, Germany. In each sample, γ-H2AX + 53BP1 colocalizing foci in 100 cells were counted. In a small number of samples, up to 200 cells were counted. To determine the average number of radiation-induced foci (RIF) per cell, the number of individual baseline foci per cell was subtracted from the number of counted foci per cell in all samples taken after administration of the radiopharmaceutical.

### Measurement of whole-body retention

External dose-rate measurements and whole-body gamma camera scans (e.cam; Siemens Healthineers, Erlangen, Germany) were performed to determine the whole-body activity retention. For the dose-rate measurements, a ceiling-mounted shielded survey meter (automess – Automation und Messtechnik GmbH, Ladenburg, Germany) fixed 2.5 m above the patient’s bed was used. At least seven measurements were taken in each patient. On the first day, measurements were taken directly (3 min up to a maximum of 38 min) after administration, prior to the first postadministration void or defaecation by the patient, and then approximately 2 h, 4 h and 7 h after administration, followed by three more measurements every 12 h on the subsequent days. All measurement data were normalised to the first initial measurement. The whole-body gamma camera scans were performed nominally 4 h, 24 h, 48 h and 96 h after administration. The last whole-body scan (96 h after administration) was missing in patients P5, P8 and P12. In patient P5, a whole-body scan approximately 68 h after administration was performed instead. Additionally, all patients underwent a SPECT/CT scan (Symbia T2 or Symbia Intevo Bold; Siemens Healthineers, Erlangen, Germany) either 24 h or 48 h after administration. To avoid the possible effects of the radiation from the CT scan on the focus count, blood samples were always taken before the SPECT/CT scan.

The count rates in counts per second were evaluated in the anterior and the posterior image of each whole-body scan using syngo.via software (Siemens Healthineers, Erlangen, Germany). The geometric mean of the background-corrected anterior and posterior count rate was then calculated. The value for the first time-point of the external dose-rate measurement was decay-corrected to the time of administration and normalised to 1, corresponding to 100% uptake. To combine the dose-rate measurements and the whole-body scan data, the count rates obtained from imaging performed 4 h after administration were normalised to the corresponding external dose-rate value 4 h after administration.

### Calculation of the absorbed doses to the blood using time-integrated activity coefficients

Triexponential and biexponential fit functions were used to describe the time curves for the activity retention in the blood and in the whole-body, respectively, in each patient. By integrating the time-activity functions over time up to the time-point *t* of the respective blood withdrawal, time-integrated activity coefficients (TIACs) for the activity concentration in the blood (*τ*_ml of bl_(*t*) in hours per millilitre) and for the whole-body (*τ*_wb_(*t*) in hours) were obtained.

The absorbed doses to the blood *D*_bl_(*t*) as a function of time were calculated as described by Eberlein et al. [14]. Additionally, the injection phase over the first 20 min with patient-specific TIAC values *τ*_ml of bl,i_ for the blood contribution and *τ*_wb,i_ for the whole-body contribution was considered in this study, as the activity was administered using a syringe pump over 20 min. The constant TIAC values *τ*_ml of bl,i_ and *τ*_wb,i_ were calculated assuming a linear uptake for −20 min < *t* < 0 until the values of *τ*_ml of bl_ (*t* = 0) and *τ*_wb_ (*t* = 0), respectively, were reached. This resulted in the following equation describing the absorbed dose to the blood:

1 $$ {D}_{\mathrm{bl}}(t)={A}_0\cdotp \left(85.3\ \frac{\mathrm{Gy}\cdotp \mathrm{ml}}{\mathrm{GBq}\cdotp \mathrm{h}}\cdotp \left({\tau}_{\mathrm{ml}\ \mathrm{of}\ \mathrm{bl}}(t)+{\tau}_{\mathrm{ml}\ \mathrm{of}\ \mathrm{bl},\mathrm{i}}\right)+\left(\frac{0.00185}{w{t}^{\frac{2}{3}}}\right)\ \frac{\mathrm{Gy}\cdotp \mathrm{k}{\mathrm{g}}^{\frac{2}{3}}}{\mathrm{GBq}\cdotp \mathrm{h}}\cdotp \left({\tau}_{\mathrm{wb}}(t)+{\tau}_{\mathrm{wb},\kern0.3em \mathrm{i}}\right)\right)\kern5pc $$

where *A*_0_ is the administered activity in gigabecquerels and *wt* is the weight of the patient in kilograms. A derivation of Eq. 1 and a detailed description of the constants therein is given in the Supplementary Material of reference [14].

The absorbed dose rate $\frac{\mathrm{d}{D}_{bl}}{\mathrm{d}t}$ is defined as the derivative of the absorbed dose to the blood *D*_bl_ (Eq. 1) over time.

### Statistical analysis

OriginPro 2017 (OriginLab Corporation) was used for data analysis, plotting and statistical evaluation. To test whether data were normally distributed, the Shapiro-Wilk test was used. For correlation analysis, Pearson correlation was applied for normally distributed datasets and Spearman correlation was applied if datasets were not normally distributed. Results were considered significant for *p* < 0.05. To test for differences between two groups of normally distributed data, *t*-tests were used. Results were considered significant for Prob>|*t*| < 0.05. The standard deviation of the focus count was calculated for each evaluated blood sample assuming a Poisson distribution of the counts in 100 cells. For the calculation of the standard deviation of the average number of RIF per cell, the standard deviation of the baseline focus count was considered additionally and error propagation was performed. Generally, the standard deviation is stated for all mean values. For the fit parameters, the standard error is given.

## Results

### Patients

A total of 16 heavily pretreated patients (P1–P16) aged between 54 and 81 years (average 70 ± 9 years) were enrolled. The mean administered activity was 5.9 ± 0.2 GBq of ^177^Lu. The demographic and clinical data of all patients are presented in Table 1.

**Table 1:** Patient demographic and clinical data

Patient ID	Age (years)	Weight (kg)	Administered activity (MBq)	Gleason score	PSA level (ng ml^−1^)		Site of metastases	Number of bone metastases^a^	Pretreatment	Response to therapy
					Before therapy	After therapy				
P1	69	78	6,020	3 + 4	48	25	Bone, lymph nodes	Disseminated	LHRH, abiraterone, enzalutamide, RTx	SD
P2	64	80	6,015	4 + 4	0.1	0.07	Lymph nodes	None	Ectomy (P + L), RTx, bicalutamide	PR
P3	68	100	5,907	4 + 3	6	6	Bone	Low	Ectomy (P), androgen blockade, CTx (D), LHRH	PD
P4	77	77	5,785	3 + 5	58	24	Bone, lymph nodes	Disseminated	RTx, bicalutamide, LHRH, CTx (D)	PR
P5	69	83	6,215	3 + 4	89	18	Bone, lymph nodes	Low	Ectomy (P + L), RTx, LHRH, bicalutamide, abiraterone, enzalutamide CTx (D)	PR
P6	70	79	5,989	5 + 4	181	683	Bone, lymph nodes	Low	Ectomy (P + L), LHRH, RTx, enzalutamide CTx (D)	PD
P7	66	85	5,758	4 + 5	151	243	Bone	Moderate	LHRH, RTx, enzalutamide	PD
P8	68	83	5,893	4 + 5	42	–	Bone, lymph nodes, liver	Low	Ectomy (P + L), LHRH, enzalutamide, CTx (D)	Death
P9	74	77	6,020	4 + 5	1,020	529	Bone, lymph nodes, liver, pleura, adrenal gland, muscle	Low	Ectomy (P + L + O), enzalutamide, RTx, abiraterone, CTx (D), ^223^Ra	PD
P10	81	71	5,703	4 + 4	252	199	Bone	Disseminated	Ectomy (P), RTx, CTx (D + C), abiraterone, LHRH	Mixed
P11	56	94	5,860	4 + 4	158	140	Bone, lymph nodes	Moderate	LHRH, RTx, enzalutamide, abiraterone, CTx (D + C), abiraterone, enzalutamide	PD
P12	65	140	5,960	4 + 4	147	144	Bone, lymph nodes	Low	LHRH, ectomy (PV), RTx, enzalutamide, CTx (C)	SD
P13	71	69	5,476	3 + 4	3,130	914	Bone, lymph nodes	Disseminated	LHRH, bicalutamide, ectomy (L), RTx, ^223^Ra, CTx (D), abiraterone	PR
P14	54	86	5,882	4 + 4	134	138	Bone	Disseminated	CTx (D), LHRH, abiraterone, RTx	Mixed
P15	80	104	5,640	4 + 4	260	174	Bone, lymph nodes	Low	Ectomy (P + O), RTx, abiraterone, CTx (D + C), enzalutamide	SD
P16	71	85	6,318	4 + 5	2,810	13,200	Bone, lymph nodes, liver	Disseminated	Enzalutamide, CTx (D), abiraterone, LHRH, enzalutamide, CTx (D + C) + denosumab	Death

*LHRH* therapy with LHRH agonists and antagonists, *RTx* external-beam radiation therapy, *CTx* chemotherapy with docetaxel (*D*) or cabazitaxel (*C*), *ectomy* surgical removal of prostate (*P*), prostate and seminal vesicles (*PV*), lymph nodes (*L*) or testicles (*O*), *PR* partial response, *SD* stable disease, *PD* progressive disease

^a^Low <10, moderate >10, disseminated >50

The pretreatments included surgery (radical prostatectomy/prostatovesiculectomy, lymphadenectomy, orchiectomy), external-beam radiation therapy, therapy with ^223^Ra dichloride, therapy with LHRH agonists and antagonists (leuprorelide, degarelix), antiandrogen therapy with abiraterone, bicalutamide, enzalutamide and flutamide, chemotherapy (docetaxel, cabazitaxel) and therapy with denosumab. All patients had a diagnostic PET/CT scan with ^68^Ga-labelled PSMA 7 to 106 days before the start of therapy and again 39 to 114 days after therapy. Additionally, all patients underwent ^99m^Tc-MAG3 scintigraphy 3 to 31 days before the start of therapy. Only in patients P2 and P4 was the MAG3 scan (with <100 MBq ^99m^Tc) performed on the day of therapy, at least 3 h before administration of ^177^Lu-labelled PSMA. Of the 16 patients, 15 (all except P2) presented with bone metastases and 12 (all except P3, P7, P10 and P14) presented with lymph node metastases. Three patients (P8, P9 and P16) additionally had liver metastases. P9 also had metastases in the pleura, adrenal gland and muscles. After the first therapy cycle, four patients showed a partial response, three patients showed stable disease, five patients showed progressive disease, and two patients showed a mixed response. Two patients (P8 and P16) died after the first therapy cycle.

### Dosimetry

A triexponential fit function was used to describe the activity retention in the blood. For describing the whole-body retention, a biexponential fit function sufficed to describe the kinetics as, in most patients, this resulted in the same curve but revealed smaller errors in the fit parameters than the triexponential fit function. With the corresponding TIAC values, which were obtained by integrating the time-activity functions over time up to the time-point *t* of the respective blood withdrawal, the absorbed doses to the blood were calculated according to Eq. 1. Consequently, the absorbed doses represent cumulative doses from the start of administration to the respective time-point *t*.

The absorbed dose to the blood directly after administration (after the 20 min injection time) was 3.2 ± 0.6 mGy on average. The average total absorbed dose to the blood, which was calculated by integrating to *t* = ∞, was 109 ± 28 mGy. The minimal total absorbed dose to the blood was 76 mGy (P3 and P11) and the maximal total absorbed dose to the blood was 164 mGy (P9). The average absorbed doses to the blood at *t*_1_, *t*_2_, *t*_3_ and *t*_4_ were 17 ± 6 mGy, 27 ± 7 mGy, 34 ± 8 mGy and 40 ± 9 mGy, respectively. For the later time-points, differences in the kinetics among the patients increased and values for the absorbed doses to the blood were no longer significantly drawn from a normally distributed population. The median absorbed doses to the blood at *t*_5_, *t*_6_ and *t*_7_ were 71 mGy (min 52 mGy, max 129 mGy), 80 mGy (min 61 mGy, max 144 mGy) and 88 mGy (min 69 mGy, max 155 mGy), respectively. At *t*_7_, in all patients except P9, more than 80% of the total absorbed dose to the blood was reached. In patient P9, the absorbed dose to the blood at this time-point was only 56% of the calculated total absorbed dose to the blood.

**Fig. 1:**
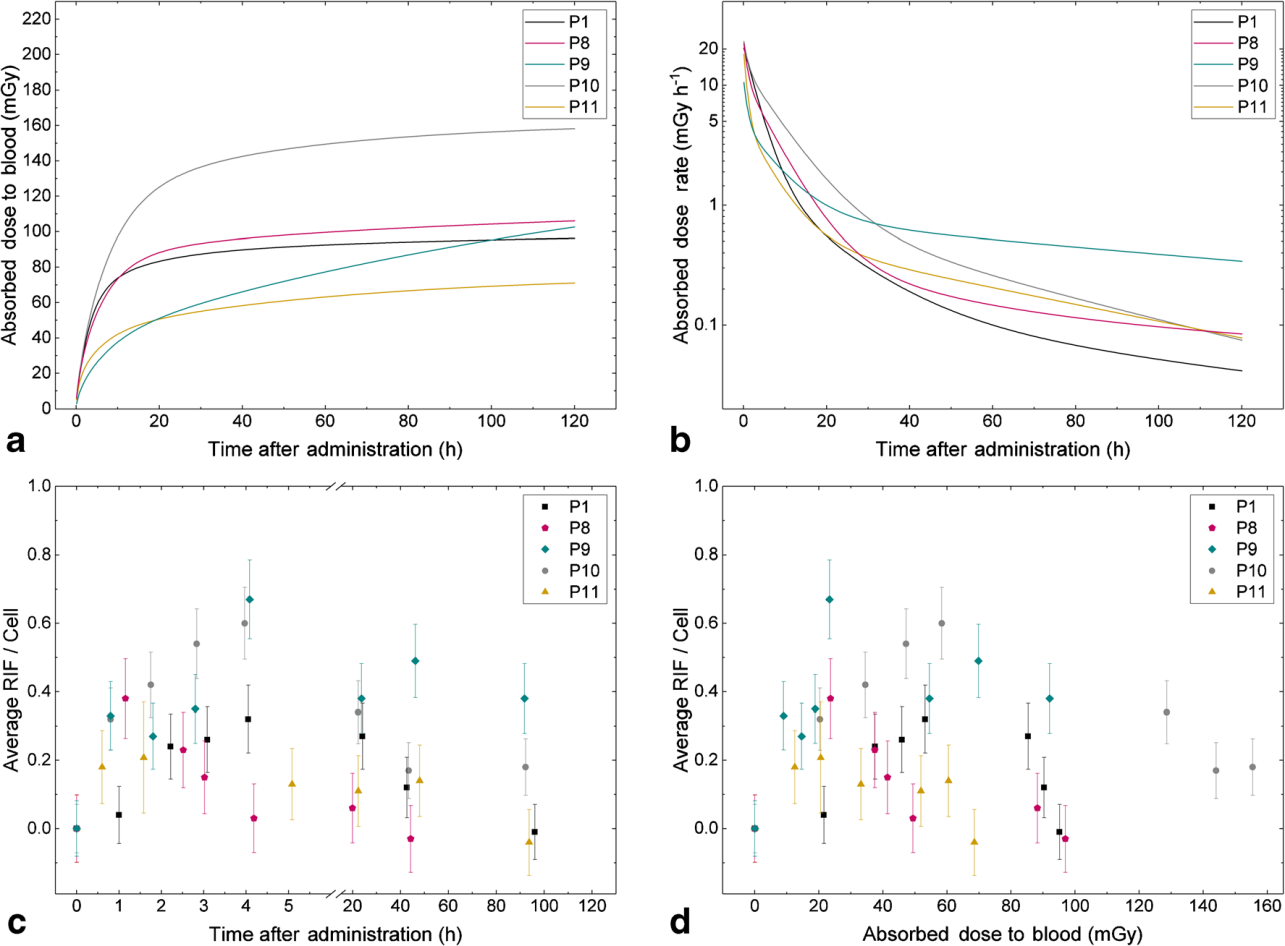
Data of five selected patients: P1 with average parameters; P8, the only patient with a decrease in radiation-induced foci (RIF) directly after the first time-point; P9, the patient with the highest dose rate at the last time-point; P10, the patient with the highest absorbed dose to the blood at the last time-point; and P11, the patient with the lowest absorbed dose to the blood at the last time-point. **a** Absorbed dose to the blood as a function of time. **b** Absorbed dose rate as a function of time. **c** Average number of RIF per cell as function of time (for better clarity a break is inserted in the *x* axis). **d** Average number of RIF per cell as function of the absorbed dose to the blood

Figure 1a shows the absorbed doses to the blood as a function of time in five selected patients. Patient P1 can be considered typical with a total absorbed dose to the blood of 103 mGy and a characteristic time-dependent curve. Patient P8 showed a similar time-dependency of the absorbed dose to the blood. Patient P9 showed a much slower and longer increase in the absorbed dose to the blood as a function of time compared with that in most of the other patients. Patients P13 and P14 also showed similar time-dependency of the absorbed dose to the blood. Patients P10 and P11 showed the maximal (155 mGy) and minimal (69 mGy) absorbed doses to the blood at the last sampling time-point, respectively.

The corresponding time-dependency of the absorbed dose rate $\frac{{\mathrm{d}D}_{\mathrm{bl}}}{\mathrm{d}t}$ is shown in Fig. 1b in the same five patients. In all patients, the average absorbed dose rates at *t*_1_, *t*_2_, *t*_3_, *t*_4_, *t*_5_, *t*_6_ and *t*_7_ were 11.59 ± 2.98 mGy h^−1^, 7.83 ± 2.11 mGy h^−1^, 6.03 ± 1.85 mGy h^−1^, 4.84 ± 1.57 mGy h^−1^, 0.72 ± 0.26 mGy h^−1^, 0.29 ± 0.14 mGy h^−1^ and 0.14 ± 0.10 mGy h^−1^, respectively. At the last sampling time-point *t*_7_, the absorbed dose rate was below 0.20 mGy h^−1^ in all patients except P9 (0.41 mGy h^−1^), P13 (0.26 mGy h^−1^) and P14 (0.25 mGy h^−1^).

### DNA damage foci

**Fig. 2:**
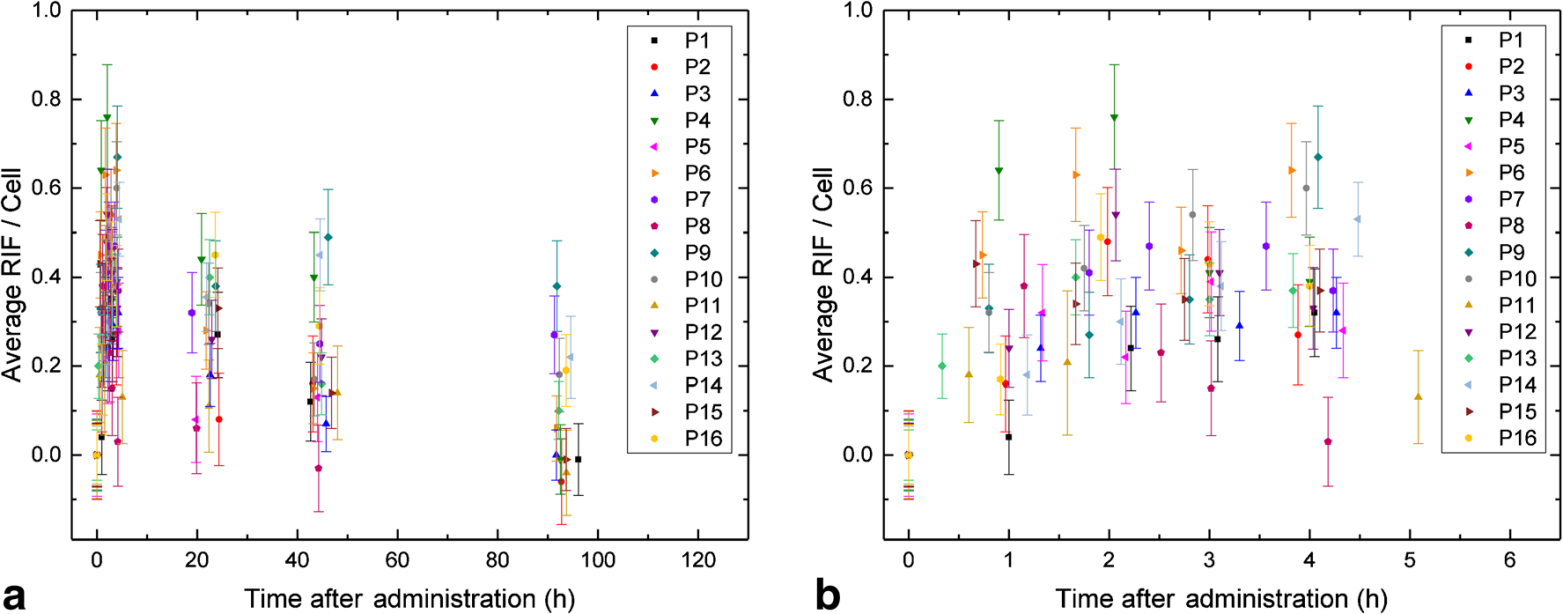
Average number of RIF per cell as a function of time after administration of ^177^Lu-PSMA. **a** Overview of all time-points. **b** Detailed view of the first five time-points (nominally 0 to 4 h after administration)

The average number of foci per cell at baseline was 0.32 ± 0.11. Paired sample *t*-tests showed that the number of foci per cell at baseline was significantly different from the values at all later time-points *t*_1_ to *t*_7_. The individual baseline value in each patient was subtracted from the number of foci for all time-points after administration to calculate the average number of RIF per cell. The average numbers of RIF per cell over all patients at *t*_1_, *t*_2_, *t*_3_, *t*_4_, *t*_5_, *t*_6_ and *t*_7_ were 0.29 ± 0.14, 0.39 ± 0.15, 0.38 ± 0.09, 0.38 ± 0.17, 0.27 ± 0.13, 0.21 ± 0.14 and 0.10 ± 0.13, respectively. The individual data for all patients are shown in Fig. 2.

Generally, the average number of RIF increased or remained constant from *t*_1_ to *t*_4_. This increase in the average number of RIF was significant from time-point *t*_1_ to *t*_2_, but not from *t*_2_ to *t*_3_ or from *t*_3_ to *t*_4_. Only in one patient (P8) was a continuous decrease in the average number of RIF observed during the first 4 h after administration, starting directly after the first time-point with 0.38 ± 0.12 RIF per cell to 0.03 ± 0.10 RIF per cell at *t*_4_ (Fig. 1c). Starting from time-point *t*_4_, the average number of RIF significantly decreased at all consecutive time-points. At the last time-point *t*_7_, 96 h after administration, in seven of 13 patients (P1, P2, P3, P4, P6, P11 and P15) the average number of foci per cell had decreased to the individual baseline values, while in six patients (P7, P9, P10, P13, P14 and P16) the number of foci was still elevated. In three patients (P7, P9 and P14), the number of RIF per cell was still above 0.2 at this time-point. As an example, the average number of RIF per cell as a function of time in P9, the patient with the highest value (0.38 ± 0.10) at *t*_7_, are shown in Fig. 1c (blue diamonds).

### Dose-dependency of the number of RIF per cell

**Fig. 3:**
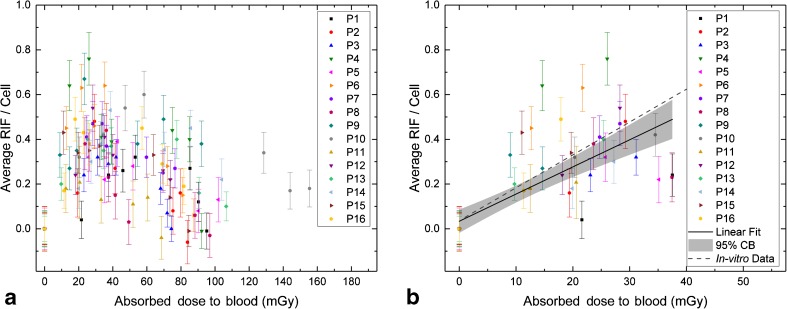
Average number of RIF per cell as a function of the absorbed dose to the blood. **a** Overview of all time-points. **b** Detailed view of the first three time-points (up to 2.6 h after administration) with a linear fit (*solid line*) to the pooled data, including a 95% confidence band (*grey area*). For comparison, the in-vitro calibration curve for ^177^Lu and ^131^I taken from reference [22] is also shown (*dashed line*)

Figure 3 shows the average number of RIF per cell as a function of the absorbed dose to the blood in all 16 patients. A linear dependency of the absorbed dose to the blood *D*_bl_ and the average number of RIF per cell was assumed for the first three time-points (*t*_0_, *t*_1_ and *t*_2_; up to 2.6 h after administration). The corresponding dose range is shown in detail in Fig. 3b. The patient data for these time-points were pooled and a linear fit to the pooled data was performed (*R*^2^ = 0.583) resulting in the linear equation:

2 $$ \mathrm{Average}\ \mathrm{RIF}\ \mathrm{per}\ \mathrm{Cell}=\left(0.0336\pm 0.0258\right)+\left(0.0122\pm 0.0015\right)\ \mathrm{mG}{\mathrm{y}}^{-1}\cdotp {D}_{\mathrm{bl}}\kern5pc $$

The slope is significantly different from zero (*t* = 8.011; Prob>|*t*| = 2.846E−10). The parameters for the lower (LCL) and upper (UCL) 95% confidence intervals are LCL = 0.009 and UCL = 0.015 for the slope and LCL = −0.018 and UCL = 0.086 for the intercept. The slope obtained in this work is 17% smaller than the slope of an in-vitro calibration curve established for ^177^Lu and ^131^I in a previous study [22]. Figure 3b shows the linear in-vitro calibration curve as a dashed line for comparison.

### Dose rate-dependency of the number of RIF per cell

**Fig. 4:**
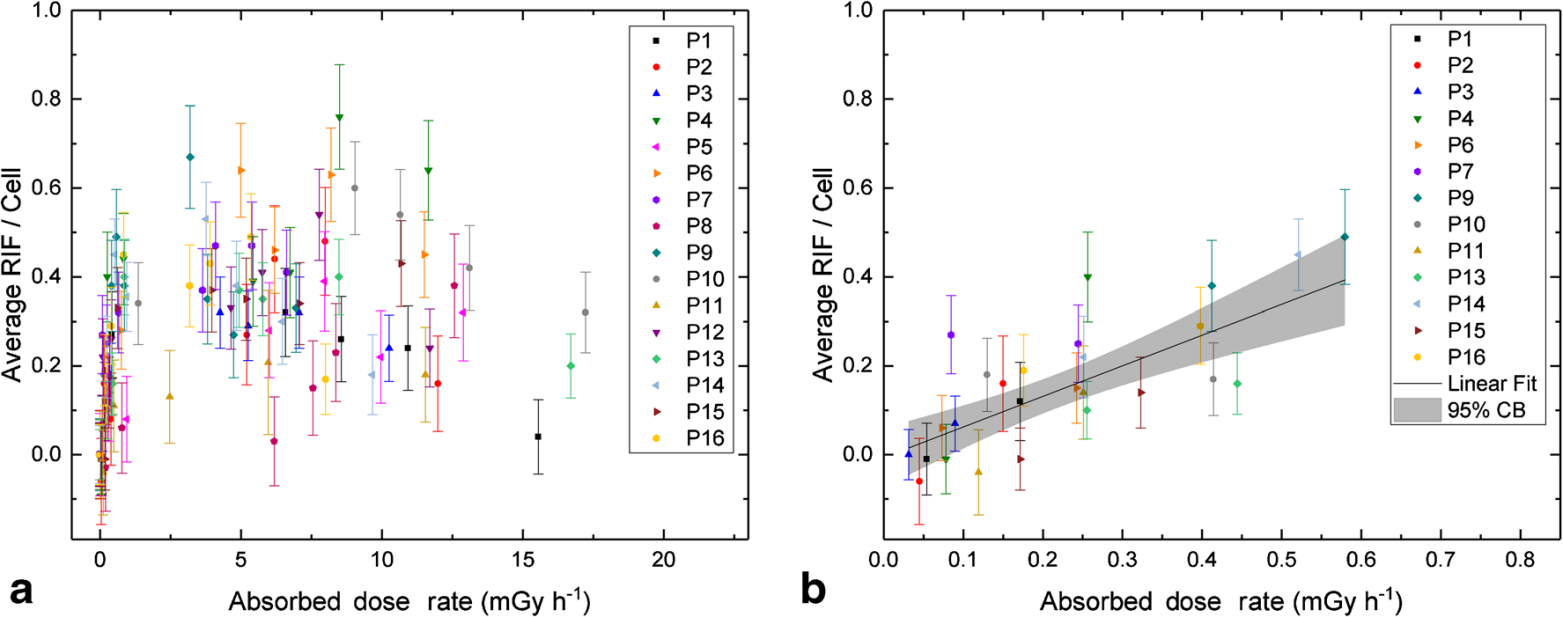
Average number of RIF per cell as a function of the absorbed dose rate. **a** Overview of all time-points. **b** Detailed view of the last two time-points (48 h and 96 h after administration) with a linear fit (*solid line*) to the pooled data, including a 95% confidence band (*grey area*). Only the 13 patients with data for both time-points were included in the graph and in the fit

Figure 4a shows the average number of RIF per cell as a function of the absorbed dose rate $\frac{\mathrm{d}{D}_{\mathrm{bl}}}{\mathrm{d}t}$ in all 16 patients. The average numbers of RIF per cell for the last two sampling time-points *t*_6_ and *t*_7_ (with absorbed dose rates of less than 0.6 mGy h^−1^) are shown for 13 patients in more detail in Fig. 4b. The three patients with a missing *t*_7_ value (P5, P8 and P12) were excluded from this graph and analysis. A fit to the pooled data revealed a linear relationship (*R*^2^ = 0.560) between the average number of RIF per cell and the absorbed dose rate in this range. The corresponding linear equation is:

3 $$ \mathrm{Average}\ \mathrm{RIF}\ \mathrm{per}\ \mathrm{Cell}=\left(-0.0066\pm 0.0326\right)+\left(0.6895\pm 0.1248\right)\ \mathrm{h}\cdotp \mathrm{mG}{\mathrm{y}}^{-1}\cdotp \frac{{\mathrm{d}D}_{\mathrm{bl}}}{\mathrm{d}t}\kern5pc $$

The slope is significantly different from zero (*t* = 5.525; Prob>|*t*| = 1.107E−5). The parameters for the 95% confidence intervals are LCL = 0.432 and UCL = 0.947 for the slope and LCL = −0.074 and UCL = 0.061 for the intercept. In accordance with this result, there was a significant correlation between the average number of RIF per cell and the absorbed dose rate at *t*_6_ (Pearson’s *r* = 0.642; *p* = 0.007) and at *t*_7_ (Pearson’s *r* = 0.705; *p* = 0.007).

### Correlations with clinical parameters

To test whether the variability in progression of RIF and the temporal course of the absorbed dose to the blood among the patients is linked to clinical parameters, correlation analysis was performed. However, because only 16 patients were included in the study, our results are based on a small amount of data. In the 13 patients with data for the last time-point *t*_7_, there was a significant positive correlation between the PSA level before therapy and the absorbed dose rate 96 h after administration (Spearman’s *ρ* = 0.819; *p* = 0.001). A weaker but still significant correlation was noted between the PSA level after therapy and the absorbed dose rate 96 h after administration (Spearman’s *ρ* = 0.659; *p* = 0.014) and between the PSA level after therapy and the average number of RIF per cell 96 h after administration (Spearman’s *ρ* = 0.619; *p* = 0.024). No significant correlations were observed between the number of bone metastases and the absorbed dose rate or the average number of RIF at *t*_7_. Parameters describing kidney function (creatinine and MAG3 before therapy) did not correlate with the absorbed dose rate or the average number of RIF at *t*_7_.

## Discussion

In the current study, we used the γ-H2AX + 53BP1 DSB focus assay to investigate the time- and dose-dependency of DNA DSB induction and repair in blood leucocytes of prostate cancer patients during their first therapy with ^177^Lu-PSMA. In general, an increase in the average number of RIF was observed in the first hours after administration of the radiopharmaceutical, followed by a decrease due to DNA repair. A similar time course was also observed in previous studies investigating γ-H2AX + 53BP1 focus induction and repair after radioiodine therapy [9, 15] and peptide receptor radionuclide therapy with ^177^Lu-labelled DOTATATE/DOTATOC [14]. In contrast to external irradiation, as in radiation therapy, internal exposure is characterized by continuous irradiation with a permanently decreasing dose rate after administration of the radionuclide. For this reason, a decrease in the number of RIF was observed at later time-points, despite the increasing absorbed dose to the blood. The disappearance of RIF at later time-points could also have been caused potentially by elimination of damaged cells. However, while high dose rate acute photon irradiation has been shown to induce apoptotic cell death in blood samples irradiated ex vivo [24, 25] and in minipigs exposed to partial body γ-irradiation with 50 Gy [23], we only occasionally noted apoptotic leucocytes in our in-vivo samples exposed to low doses and low dose rates. Therefore, we consider it unlikely that a preferential elimination of damaged cells influenced the repair time courses observed.

In the present study, the mean total absorbed dose to the blood was 109 ± 29 mGy after administration of 5.9 ± 0.2 GBq. The median absorbed dose to the blood 48 h after administration was 80 mGy (min 61 mGy; max 144 mGy). This is comparable with the results of Eberlein et al. who observed an absorbed dose to the blood of 79 ± 16 mGy 48 h after administration of 7.2 ± 0.4 GBq ^177^Lu-labelled DOTATATE/DOTATOC [14]. The total absorbed dose to the blood could not be calculated by Eberlein et al. since no data were available for time-points >48 h after administration [14].

Up to 2.6 h after administration (time-points *t*_0_, *t*_1_ and *t*_2_), there was a linear relationship between the average number of RIF per cell and the absorbed dose to the blood. A linear increase in the average number of RIF was also observed in the first 2 h after administration of ^131^I [15] and up to 5 h after administration of ^177^Lu-DOTATATE/DOTATOC [14]. As in these previous studies, the slope of the linear curve obtained in this investigation was less than the slope of the in-vitro calibration curve established for ^177^Lu and ^131^I by Eberlein et al. [22]. Here, the difference to the in-vitro slopes was 17%, while in the patient studies investigating the induction of RIF after radioiodine therapy and therapy with ^177^Lu-DOTATATE/DOTATOC, the differences between the slopes of the in-vivo and the in-vitro data were 20% and 14%, respectively [14, 15]. The slightly shallower slope observed in all patient studies can be explained by the onset of DNA repair. Another reason for the differences between the slopes may be the greater inaccuracy in the calculation of the absorbed doses for the early time-points, as the first measurement for activity quantification was taken not earlier than 1 h after administration. Finally, it is possible that the difference in cellular environment (redox state) between the systemic in-vivo exposure and the “artificial” ex-vivo irradiation in blood tubes may have led to a lower induction of DSBs in patients.

While the average number of RIF per cell correlated with the absorbed dose to the blood during the first hours after administration of the radiopharmaceutical, we found that the absorbed dose rate was the determining factor for RIF progression at later time-points *t*_6_ and *t*_7_ (48 h and 96 h after administration). During the first hours after administration, no correlation was observed between the absorbed dose rate and the average number of RIF per cell. For the late time-points *t*_6_ and *t*_7_, however, there was a linear relationship between the dose rate and the average number of RIF per cell. This indicates that patients with relatively high absorbed dose rates exhibit either slower focus repair kinetics or an increased rate of focus induction even at late time-points. An absorbed dose rate-dependency of RIF was also observed by Lassmann et al. in patients with differentiated thyroid cancer undergoing radioiodine therapy [9]. In other studies investigating γ-H2AX foci after radionuclide therapy [14, 15, 17, 18], an absorbed dose rate-dependency of RIF was not considered.

For the latest time-points in particular, we also observed a high variability in the data among the patients. In seven of 13 patients, the number of foci per cell 96 h after administration had decreased to the baseline values. The other patients still showed elevated numbers of foci correlating with higher absorbed dose rates at this time-point.

To find a link between medical parameters and the variable effectiveness of DNA DSB repair among the patients, we compared the RIF and dose rate data with clinical endpoints and found significant correlations with PSA levels. However, for the correlation analysis only the data from 13 patients with different pretreatments could be included. To validate the current findings and to exclude possible confounding factors, more patients and data are needed.

Although the general pattern of the time- and dose-dependencies of RIF induction were similar to those found in previous studies [14, 15], we observed a higher variability among the patients overall in this study, which is reflected in a wider distribution of the foci data and a weaker correlation for the linear fit as a function of the absorbed dose to the blood for the first data points. A possible reason for this could be the comparatively longer medical history and the individual variability in the extensive pretreatments of the patients. Other possible causes of the variability among the patients are variations in the absorbed dose delivery directly after administration and differences in individual repair kinetics. In particular at late time-points, repair kinetics are influenced by varying dose rates. In order to link the dose-dependency in the first hours after administration and the dose rate-dependency for the last sampling time-points, patient-specific kinetic modelling, taking into account the dose-dependency of focus induction and repair, may be applied in future studies.

### Conclusion

This is the first study of the induction and persistence of DSB DNA damage combined with internal dosimetry after therapy with ^177^Lu-PSMA. The general pattern of the time-dependent induction and disappearance of RIF follows that of other radionuclide therapies. In some patients, RIF had not disappeared completely 96 h after administration, which can be explained by an above average absorbed dose rate. In the majority of patients, however, the DSBs induced in blood leucocytes were effectively repaired. The correlation with clinical findings needs further research in a larger number of patients.
